# Drivers of Below‐Cutoff Scores on Bedside Cognitive Screening in Cognitively Normal Ethnoracially Diverse Adults

**DOI:** 10.1002/alz70857_105661

**Published:** 2025-12-25

**Authors:** Paula Aduen, John A. Lucas, Christian Lachner, Yoav D Piura, Leah Schecter, Minerva M. Carrasquillo, Richard O White, Neill R. Graff‐Radford, Gregory S Day, Manoj K Jain

**Affiliations:** ^1^ Mayo Clinic in Florida, Jacksonville, FL, USA

## Abstract

**Background:**

Bedside cognitive screening tools intend to reliably and efficiently detect cognitive impairment in research, clinical, and community settings. Black American (BA) and Hispanic/Latino (H/L) adults obtain lower performance on widely used screeners, largely driven by demographics and structural and social determinants of health (SSDoH). This study characterized the performance of the Montreal Cognitive Assessment (MoCA) performance in a cohort of cognitively normal ethnoracially diverse adults while considering cohort‐specific factors that contributed to misclassification of impairment.

**Method:**

Cognitively normal BA and H/L participants completed a comprehensive neurological assessment, neuropsychological testing, and MRI and amyloid‐ and tau‐PET imaging as part of a longitudinal study of memory and aging at Mayo Clinic in Florida. Demographically adjusted MoCA cutoffs were applied to BA (MoCA ≤ 22) and WH/L (MoCA ≤ 24) subgroups. Hierarchical binary logistic regression assessed whether amyloid burden (Centiloid value) improved prediction of scoring below demographically adjusted MoCA cutoffs beyond demographic/SSDoH factors, including age, education, and Area Deprivation Index (ADI).

**Result:**

Participants included 105 (47 WH/L, 58 BA/AA) cognitively normal adults (mean age = 64.71 years, SD = 8.91; 62.9% female) with average of 16.07 years of education (SD=2.35) and mild‐to‐moderate neighborhood disadvantage (mean ADI=47.60, SD=23.77). 40% of H/L participants (MoCA (≤ 24) and 17% of BA participants (MoCa ≤22) scored below demographically adjusted MoCA cut‐offs. For the H/L subgroup, the best fitting model included age, education, ADI, and amyloid burden (*R* ^2^ = 0 .43, χ^2^ = 10.98, *p* =  0.03). Age (OR=1.21, 95% CI: 0.986 – 1.487) and amyloid burden (OR = 0.83, 95% CI: 0.67 – 1.03) approached statistical significance as individual predictors (*p* = 0.06, p*=*0.09). In the BA/AA subgroup, the best fitting model included age, education and ADI only (*R* ^2^ = 0.47, χ^2^ = 9.06, *p* =  0.02), with education approaching significance as an individual predictor (OR=0.46, 95% CI: 0.21 – 1.01, *p* = 0.05).

**Conclusion:**

Sociodemographic factors continued to drive low MoCA performance in cognitively normal individuals despite applying demographically adjusted cut‐off scores by subgroup. These factors differentially impacted performance among BA/AA and WH/L participants. Careful consideration of these factors is warranted to mitigate risk of overdiagnosing impairment based on cognitive screening across clinical and research settings.

